# Age-specific malaria seroprevalence rates: a cross-sectional analysis of malaria transmission in the Ouest and Sud-Est departments of Haiti

**DOI:** 10.1186/1475-2875-13-361

**Published:** 2014-09-14

**Authors:** Michael E von Fricken, Thomas A Weppelmann, Brandon Lam, Will T Eaton, Laura Schick, Roseline Masse, Madsen V Beau De Rochars, Alexandre Existe, Joseph Larkin, Bernard A Okech

**Affiliations:** Emerging Pathogens Institute, University of Florida, 2055 Mowry Road, Gainesville, FL 32611 USA; Department of Environmental and Global Health, University of Florida, Gainesville, FL 32610 USA; Department of Microbiology and Cell Science, University of Florida, Gainesville, FL 32611 USA; Community Coalition for Haiti, Portail Leogane Clinic, Jacmel, Haiti; Christianville Foundation, Christianville Boulevard Mareshall, Gressier, Haiti; Department of Health Services Research Management and Policy, University of Florida, Gainesville, FL 32611 USA; Laboratoire National de Santé Publique, Ministère de la Santé Publique et de la Population, Port-au-Prince, Haiti

**Keywords:** Malaria, Haiti, *Plasmodium falciparum*, AMA-1, MSP-1_19_, Serology, SCR

## Abstract

**Background:**

Malaria transmission continues to occur in Haiti, with 25,423 confirmed cases of *Plasmodium falciparum* and 161,236 suspected infections reported in 2012. At low prevalence levels, passive surveillance measures, which rely primarily on reports from health systems, becomes less appropriate for capturing annual malaria incidence. To improve understanding of malaria transmission in Haiti, participants from the Ouest and Sud-Est departments were screened using a highly sensitive enzyme-linked immunosorbent assay (ELISA).

**Methods:**

Between February and May 2013, samples were collected from four different sites including a rural community, two schools, and a clinic located in the Ouest and Sud-Est departments of Haiti. A total of 815 serum samples were screened for malaria antibodies using an indirect ELISA coated with vaccine candidates apical membrane antigen (AMA-1) and merozoite surface protein-1 (MSP-1_19_). The classification of previous exposure was established by using a threshold value that fell three standard deviations above the mean absorbance for suspected seronegative population members (OD of 0.32 and 0.26 for AMA-1 and MSP-1, respectively). The observed seroprevalence values were used to fit a modified reverse catalytic model to yield estimates of seroconversion rates.

**Results:**

Of the samples screened, 172 of 815 (21.1%) were AMA-1 positive, 179 of 759 (23.6%) were MSP-1_19_ positive, and 247 of 815 (30.3%) were positive for either AMA-1 or MSP-1; indicating rates of previous infections between 21.1% and 30.3%. Not surprisingly, age was highly associated with the likelihood of previous infection (p-value <0.001). After stratification by age, the estimated seroconversion rate indicated that the annual malaria transmission in the Ouest and Sud-Est department is approximately 2.5% (95% CI SCR: 2.2%, 2.8%).

**Conclusions:**

These findings suggest that despite the absence of sustained malaria control efforts in Haiti, transmission has remained relatively low over multiple decades. Elimination in Haiti appears to be feasible; however, surveillance must continue to be strengthened in order to respond to areas with high transmission and measure the impact of future interventions.

## Background

Over the past decade there has been a renewed interest in eliminating malaria from the island of Hispaniola, with a bi-national strategy recently adopted between the Dominican Republic and Haiti to eliminate malaria by 2020 [1]. Recent reports of emerging chloroquine resistance in Haiti [2], coupled with increased international aid, present a time sensitive window in which malaria control efforts should be scaled up, before treatment strategies must switch to more expensive combination therapies [3]. Furthermore, only one species of malaria parasite is present in Haiti, *Plasmodium falciparum,* and the principal mosquito responsible for malaria transmission, *Anopheles albimanus,* is primarily zoophilic making it a poor vector of disease [1]. Finally there is little chance of malaria being reintroduced into Haiti once it has been successfully eliminated [4].

Although transmission continues to occur in Haiti, with 25,423 confirmed cases and 161,236 suspected infections reported in 2012 [5], findings from a 2012 country wide cross-sectional survey administered by Population Services International suggest parasite prevalence rates to be <1% [1]. However, focal transmission has been documented by other studies, with parasite rates in the Artibonite Valley of 3.1% [6], and parasite rates ranging from 0-34% in the Sud-Est Department [7], indicating persistent and heterogeneous malaria transmission.

As Haiti gears up for malaria elimination, obtaining sensitive measurements of malaria transmission will be crucial to monitoring the impact of control efforts adopted to achieve this goal [8]. In low transmission settings, there is a tendency to rely on passive malaria surveillance over active surveillance due to budgetary constraints; however, passive surveillance is not as sensitive at accurately capturing malaria incidence, especially in areas with poor health infrastructure like Haiti. To overcome this difficultly, serological markers of malaria have been used to determine malaria exposure rates in low transmission settings, allowing researchers to estimate seroconversion rates (SCR) by modelling the age specific seroprevalence [9–15]. Recently, a study by Arnold *et al.* examined cross-sectional and longitudinal data from 1991-1998 using merozoite surface protein-1_19_ (MSP-1), and found the SCR to be roughly 2.3% in Leogane, which is located in the Ouest department of Haiti [12]. Estimating malaria transmission by measuring long-lasting antibody responses generated from previous malaria infections also allows the investigation of long-term trends without the estimated seroconversion rates being skewed by seasonal transmission, which is appropriate in this setting since the endemic-epidemic state of malaria coincides closely with rainfall patterns in Haiti [10, 16].

The purpose of this study was to provide valuable information on current trends in malaria transmission in the Ouest and Sud-Est departments of Haiti by analysing data collected in 2013 with ELISA techniques employing more than one *P. falciparum* specific antigen. This data adds to the current body of literature on malaria in Haiti, while providing policy-makers baseline information on malaria transmission rates in these regions that support the rationale for malaria elimination in Haiti.

## Methods

### Study location and enrollment

**Figure 1:**
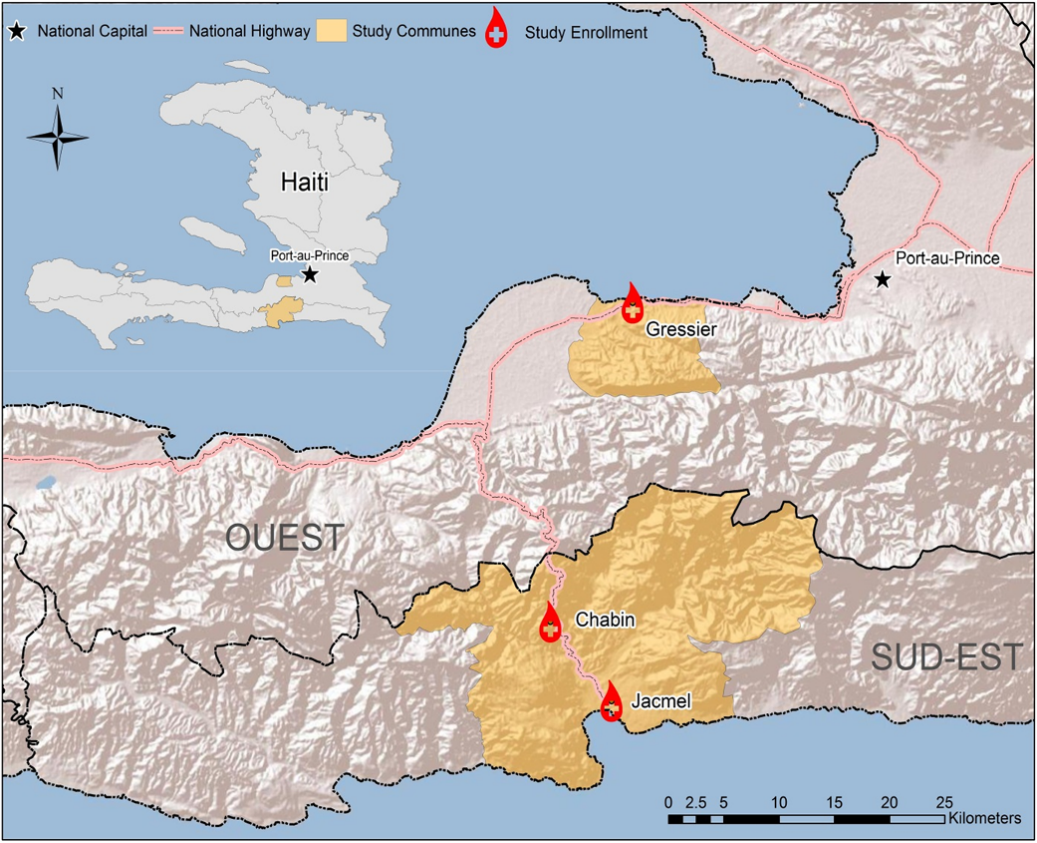
**Map of Haiti with the location of the study enrollment sites.** The four study enrollment sites located inside the Ouest and Sud-Est Departments of Haiti in the communes of Gressier and Jacmel. Participants were enrolled from Christianville School in Gressier and from Hosana Baptist School and Portail Leogane Clinic, and the rural community of Chabin in Jacmel. Along with an inset of the entire country of Haiti, the enrollment sites (red circles) appear relative to the study commune (yellow), the national capital (star) and national highway systems (pink lines).

The samples analysed in this study were collected from four sites located in the Ouest and Sud-Est department of Haiti in the communes of Gressier and Jacmel, between February and May 2013. A map of Haiti including the enrollment locations, study communes, and departments is presented in Figure 1. Enrollment was based on convenience sampling from both clinical and non-clinical settings, as part of a larger study on host protective genetic factors [17]. Study sites included a rural community, two schools, and a clinic located in the Ouest and Sud-Est departments of Haiti, with participation open to all individuals attending each site. Healthy children were enrolled from the Christianville School in Gressier and from the Hossana Baptist School in Jacmel. Patients and healthy family members attending Portail Leogane Clinic in Jacmel were enrolled on a voluntary basis. Individuals from Chabin were enrolled from community members seeking general health services at a mobile clinic. Participants at each location were given opportunities to ask the enrolling physicians questions during information sessions prior to consent. After obtaining consent from participants or their guardian, local clinicians collected approximately 3 ml of blood by venipuncture into serum separation tubes from participants, which were centrifuged immediately at 6,000 RPM for two minutes after collection was complete. All serum samples were stored at -80°C in the University of Florida field laboratory in Gressier, until shipment to the Emerging Pathogens Institute, in Gainesville, FL, for analysis and storage. Serum samples were collected from a total of 823 participants between the ages of two and 80. Malaria infected participants (5/823), determined via rapid diagnostic test (RDT) were excluded from analysis to reduce the effect of positive individuals seeking treatment for current infections. Participants missing age data (3/823) were also excluded, resulting in a final sample size of 815 (483 females and 332 males). Of the 815 samples analysed, all were screened for previous exposure using the antigen AMA-1, but only 759/815 samples were screened using the MSP-1, due to limited amounts of serum from some participants.

### Ethical approval

Ethical approval to conduct this research was obtained from the Haitian-based Ethical Review Committee, the University of Florida Institutional Review Board, and the Office of Research Protections, United States Army Medical Research and Materials Command.

### ELISA protocol and procedures

Serum samples were screened for antibodies against AMA-1 and MSP-1_19_ using an indirect enzyme-linked immunosorbent assay (ELISA). Serum samples of subjects were diluted in 5% non-fat skim milk in phosphate buffered saline (NFSM-PBS). ELISA plates were coated in duplicate with the respective antigen diluted in 5% NFSM-PBS to a final concentration of 1 ml/ml for AMA-1 and 0.5 ml/ml for MSP-1, before overnight incubation at 4°C. The next day, antigen was removed and plates were washed five times with 0.05% tween-20 in PBS-K and then blocked for one hour with 5% NFSM-PBS to reduce non-selective binding. Following additional wash, diluted serum samples as well as positive and negative control sera were plated in duplicate and incubated for two hours at 4°C. Horseradish peroxidase conjugated rabbit anti-human IgG secondary antibody was diluted 1:1,000 in 5% NFSM-PBS and added to the plate. After one hour, plates were washed seven times and treated with 3,3’, 5,5’-tetramethylbenzidine (TMB) substrate solution in the dark for 20 minutes to allow sufficient colour development and stopped with 2 M sulfuric acid.

### Determination of seropositive and seronegative population members

**Figure 2:**
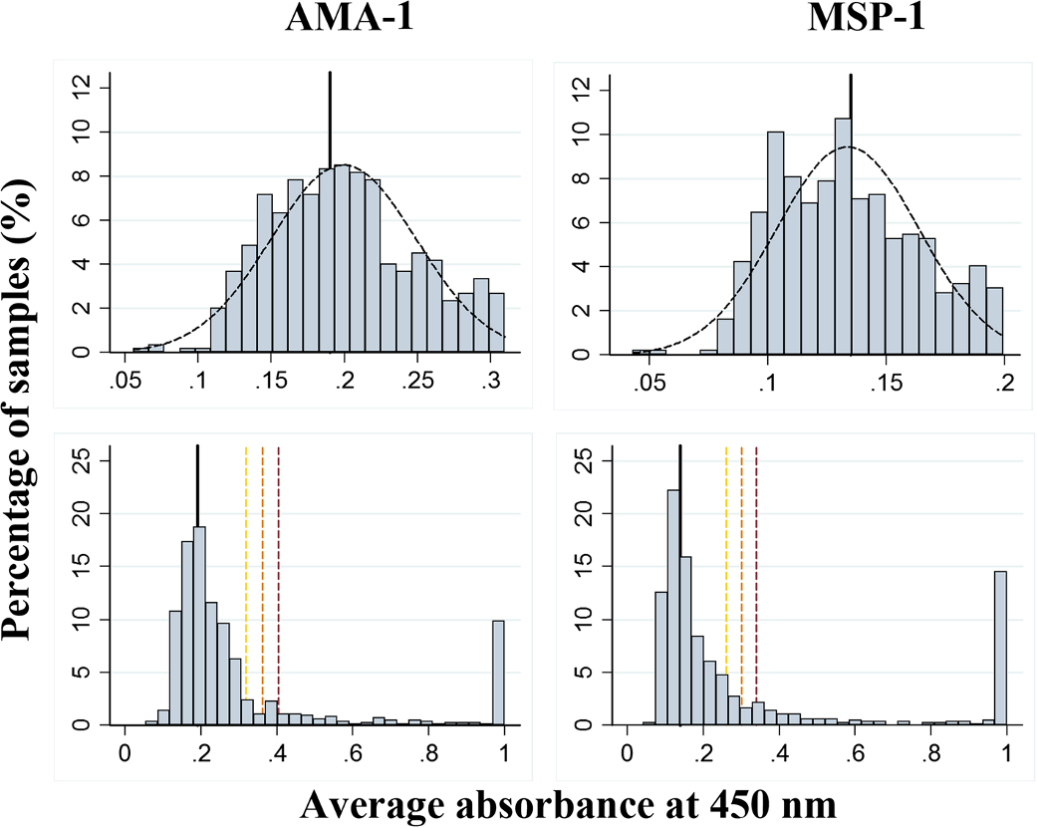
**Distribution of ELISA responses in absorbance units (λ = 450 nm).** The distribution of ELISA responses from the study participants in absorbance units (at 450 nm) appear for AMA-1 and MSP-1 on the left and right panels of Figure 2, respectively. The upper panels show the histograms of the suspected seronegative ELISA results overlaid with a normal distribution function. The sample mean (thick black line) and sample standard deviation of these functions were used to determine minimum absorbance values (thresholds) for the classification of a sample as seropositive using the sample mean for the suspected negative population members plus three to five sample standard deviations (gold, orange, and red dashed lines).

After measurement of the samples in duplicate, the average absorbance at 450 nm was used to determine the thresholds for the classification of a sample as seropositive or seronegative. Given the low incidence and relatively large sample size for each antigen (n = 815 or 759), the seronegative population responses for AMA-1 and MSP-1 should follow a normal distribution function with sample mean  and sample standard deviation . This behaviour is presented in Figure 2, where the suspected seronegative populations approximated normal distributions with  and  for AMA-1 and MSP-1, respectively. Thresholds for positive AMA-1 and MSP-1 responses were classified by the addition of three, four, and five sample standard deviations to the sample mean, such that ≥ 99.7% of seronegative population members would not be classified as seropositive. The resulting thresholds using three, four, and five, sample deviations for AMA-1 were 0.319, 0.362, 0.405 and 0.260, 0.30, 0.340 for MSP-1. Responses above three standard deviations of the mean suspected average were considered seropositive, given the minimal impact using more conservative thresholds had on the estimated prevalence (see Discussion).

### Estimation of seroconversion rates from cross-sectional data

The observed cross-sectional seroprevalence was used to estimate the seroconversion rate using a method similar to those previously described [11, 18]. Briefly, an age specific seroconversion model was fit to the prevalence of AMA-1, MSP-1, and AMA-1 or MSP-1 seropositive population members using all participants (aged 2 to 80) and separately for participants less than 20 years of age to estimate the rate of seroconversion (λ). Participants were separated into “age classes” to depict aggregated seroprevalence, with wider age ranges used in older groups, due to the lower number of participants over the age of 20. Since the AMA-1 and MSP-1 responses are long-lasting and the estimation of reversion rates has been suggested to be unreliable with cross-sectional data, a reversion rate (ρ) of zero was used for the final analysis [13], however non zero reversion rates were also explored. Without seroreversion, the probability of infection (prevalence) at age *x* was modeled using the equation *P*(*x*) = [*1* ‒ *exp*(-*λ* * *x*)]*.* When seroreversion was included in the model, the probability at age *x* was modeled using the equation *P*(*x*) = *λ*/(*λ* + *ρ*) [*1* ‒ *exp*(-(*λ* + *ρ*)*x*)]*.* The functions were optimized (using R) to give estimated seroconversion/reversion rates  and , as well as their standard errors for the calculation of 95% confidence intervals for  and . Odds ratios for the probability of having a previous exposure, as determined by a positive ELISA response, were calculated using a simple logistic regression by age category.

## Results

### Estimation of seroprevalence using AMA-1 and MSP-1

Characteristics of the resulting study population can be found in Table 1.

**Table 1:** **Study population characteristics by site of enrollment**

	Demographic factors			Participants given ELISA		
Site of enrollment	Size	Age	Gender	AMA-1	MSP-1	Combined
	(N)	Years	% Male	No. tests	No. tests	No. tests
Christianville school	510	12.2	41.4	510	461	510
Hosana Baptist school	102	8.29	52	102	102	102
Portail Leogane clinic	72	28.9	29.2	72	72	72
Chabin community	131	29.8	35.9	131	124	131
Total	815	16.1	40.7	815	759	815

The total number of total samples, average age in years, percentage of male participants, and the number of participants who were screened using an ELISA with AMA-1, MSP-1, or either AMA-1 or MSP-1 antigens are listed by study enrollment site.

**Figure 3:**
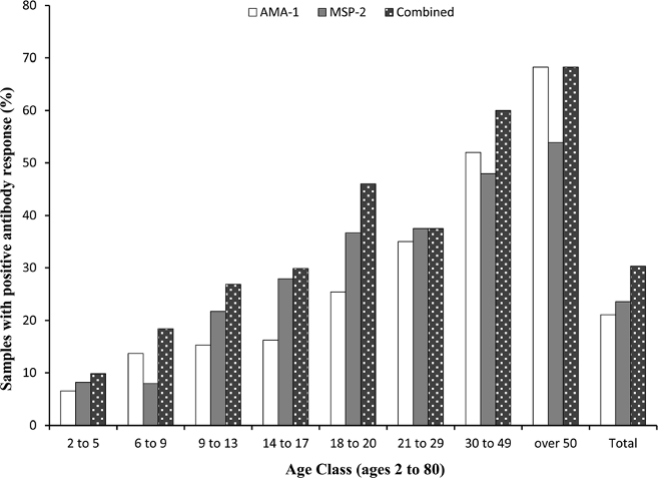
**Seroprevalence by age class for participants ranging from 2 to 80 years of age as determined by ELISA using AMA-1 and MSP-1 antigens.** The prevalence of samples that had an response to the AMA-1 or MSP-1 antigens are presented by age class for those classified as being seropositive with AMA-1 (white), MSP-1 (charcoal), or either (black dotted) antibodies.

Using the average absorbance from each serum sample and a threshold of three standard deviations from the sample mean to indicate the presence of AMA-1 or MSP-1 antibodies, 172 of 815 (21.1%) had the presence of AMA-1 antibodies, 179 of 759 (23.6%) had the presence of MSP-1 antibodies, and 247 of 815 (30.3%) had the presence of either AMA-1 or MSP-1 antibodies. Using thresholds from three to five standard deviations gave similar estimates of seroprevalence that ranged from 16.3% to 21.1% for AMA-1, 17.4% to 23.6% for MSP-1 and 24.0% to 30.3% for either AMA-1 or MSP-1. The seroprevalence by age group (ranging from 2 to 80 years) is presented in Table 2 and Figure 3.

**Figure 4:**
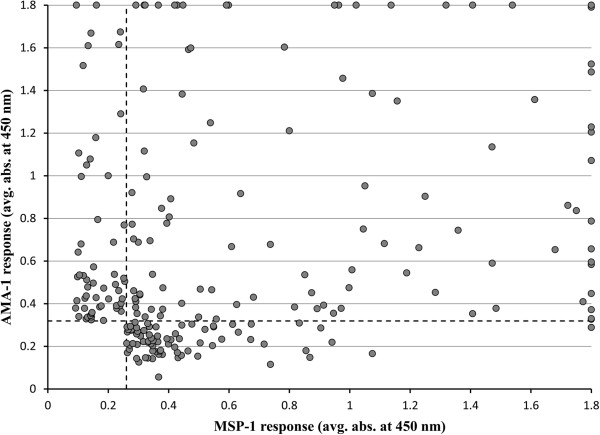
**Comparison of samples with positive AMA-1 or MSP-1 responses.** The scatterplot of AMA-1 and MSP-1 responses (average absorbance at 450 nm) is shown along with the reference lines denoting positive AMA-1 or MSP-1 responses (dotted lines) using the threshold absorbance values for previous infection of 0.319 and 0.260 absorbance units, respectively. Samples were positive for AMA-1 and MSP-1 (region I), MSP-1 positive and AMA-1 negative (region II), AMA-1 positive and MSP-1 negative (region III), or negative for both AMA-1 and MSP-1 (region IV). For clarity, samples with an absorbance value above 1.8 AU are shown as having a maximum value of 1.8 AU and those with no positive response to either antigen (region IV) are not shown.

As expected, the prevalence of seropositive participants increases with participant age. The prevalence of previous infections determine by AMA-1 response was not significantly different (p value > 0.05) between age classes under 20 years of age. However compared to participants younger than 20 years of age, the likelihood of previous infection in those over 20 years of age was 6.0 times higher (95% CI OR 4.03, 8.99). For MSP-1 responses, differences in prevalence compared to the youngest age class were significant (p value = 0.023) beginning at the third age class (9 to 13 years of age), with those over 20 years of age having 3.7 times the likelihood of previous infection (O.R. – 3.7; 95% CI = 2.48, 5.53). Of participants who were tested with both AMA-1 and MSP-1 (n = 759), 104 were classified as seropositive using both antigens (13.7%), 75 were MSP-1 positive and AMA-1 negative (9.9%), 57 were AMA-1 positive and MSP-1 negative (7.5%), and 523 were negative for both AMA-1 and MSP-1 (68.9%). The distribution of positive ELISA responses for AMA-1 or MSP-1 antigens appears graphically in Figure 4, with regions I, II, III, and IV representing the average absorbance value for the response to the AMA-1 and MSP-1 antigens for samples with AMA-1(+)/MSP-1(+) results, AMA-1(-)/MSP-1 (+) results, AMA-1(+)/MSP-1(-) results, and AMA-1 (-)/MSP-1(-) results, respectively.

**Table 2:** **Number and prevalence of seropositive participants by age class**

Age class	AMA-1			MSP-1			Combined		
(years)	n	No. Pos.	% Pos.	n	No. Pos.	% Pos.	N	No. Pos.	% Pos.
2 to 5	61	4	6.6	61	5	8.2	61	6	9.8
6 to 9	190	26	13.7	189	15	7.9	190	35	18.4
9 to 13	216	33	15.3	193	42	21.8	216	58	26.9
14 to 17	154	25	16.2	129	36	27.9	154	46	29.9
18 to 20	63	16	25.4	60	22	36.7	63	29	46.0
21 to 29	40	14	35.0	40	15	37.5	40	15	37.5
30 to 49	50	26	52.0	48	23	47.9	50	30	60.0
over 50	41	28	68.3	39	21	53.8	41	28	68.3
Total	815	172	21.1	759	179	23.6	815	247	30.3

The number of total samples, positive results, and prevalence for each age class appear for participants with positive ELISA results toward AMA-1, MSP-1, or either AMA-1 or MSP-1 antigens.

### Estimation of seroconversion rates for AMA-1 and MSP-1

**Figure 5:**
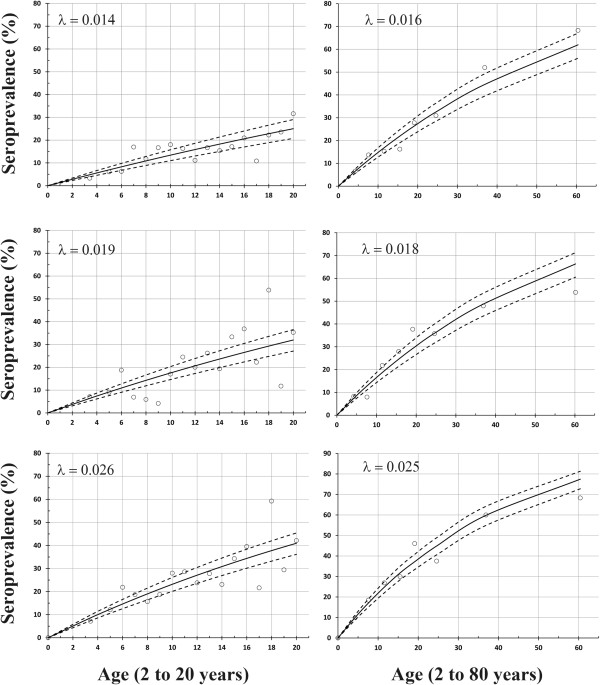
**Seroprevalence estimates for the presence of AMA-1 or MSP-1 antibodies by age for the entire study population.** The actual seroprevalence for AMA-1 and MSP-1 antibodies (circles) appear by age along with the probability of infection in each age class for AMA-1 and MSP-1 (black lines) and the respective 95% confidence limits (dotted lines), derived from the model estimated seroconversion rate (*λ*). The top, middle, and lower panels show the incremental increases in seroprevalence for AMA-1, MSP-1, and either AMA-1 or MSP-1 with age, respectively. The left panels show the model fits using data from participants 2 years to 20 years of age, while the right panels show the model fits using the entire data set including participants from 2 to 80 years.

The prevalence and the estimated probability of previous infection as determined by AMA-1 and MSP-1 antigen response are presented in Figure 5 for the entire study population (2 to 80 years) and those less than 20 years of age. The estimated SCR for AMA-1 (top panel) from the entire study population was 0.016 (SCR – 0.016; 95% CI = 0.013, 0.018) and 0.014 (SCR – 0.014; 95% CI = 0.012, 0.017) when fit to data from participants 20 years or younger. For MSP-1 (middle panel), the estimated SCR from the entire study population was 0.018 (SCR – 0.018; 95% CI = 0.016, 0.021) and 0.019 (SCR – 0.019; 95% CI = 0.015, 0.022) for participants 20 years or younger. Using AMA-1 or MSP-1 to define seropositive participants (bottom panel), the estimated SCR from the entire study population was 0.025 (SCR – 0.025; 95% CI = 0.022, 0.028) and 0.026 (SCR – 0.026; 95% CI = 0.022, 0.302) for participants 20 years or younger. When a non-zero seroreversion rate (SRR) was used to model the seroprevalence data from all participants aged 2 to 80 years of age, the following conversion and reversion rates were derived: 0.014 (95% CI  0.011, 0.017) and -0.006 (SRR– 0.006; 95% CI = -0.016, 0.003) for AMA-1 responses, 0.0204 (SCR – 0.204; 95% CI = 0.016, 0.025) and 0.008 (SRR – 0.008; 95% CI = -0.005, 0.022) for MSP-1 responses, and 0.0273 (SCR – 0.0273; 95% CI = 0.022, 0.032) and 0.007 (SRR – 0.007; 95% CI = -0.003, 0.018) for either an AMA-1 or MSP-1 response.

## Discussion

In an effort to meet the island wide goal of malaria elimination by 2020, the gametocidal drug primaquine (PQ), was added to the malaria national treatment policy for Haiti in 2010 [1]. This treatment policy change places Haiti in a unique position to monitor and quantify the impact single dose PQ administration has on *P. falciparum* transmission, which could hold valuable information on PQ tolerance and malaria elimination strategies abroad. Findings suggest that these regions have experience a relatively low and constant state of *P. falciparum* transmission, given the stable increase in seroprevalence by age observed in this study. In samples that had positive responses to either AMA-1 or MSP-1, the estimated SCR of 2.5% (95% CI  2.2%, 2.8%) from this study is slightly higher than the <1% prevalence rate estimate by PSI in 2012 [3]. However, when the seroconversion rates were determined individually from a positive AMA-1 or MSP-1 response, the estimated SCR decreased to 1.6% (95% CI  1.3%, 1.8%) and 1.8% (95% CI  1.6%, 2.1%) for AMA-1 and MSP-1, respectively. The differences in SCR estimates could be a result in variation in individual antibody responses, as suggested by Figure 4, where some seropositive respondents have strong responses to only a single antigen (regions II and III). Trends were also observed in the antibody responses after stratification for age, which could indicate that the duration of AMA-1 and MSP-1 antibody titers are different, as previously suggested [19]. In Figure 3, in 4 out of 5 age groups below 20 years of age the seroprevalence of MSP-1 was higher than the seroprevalence of AMA-1, whereas 2 of 3 of the age groups above 20 show higher seroprevalence of AMA-1 compared to MSP-1. However, in this sample the likelihood of participants having a strong AMA-1 response ( > 0.5 AU) and a MSP-1 response below the threshold (Figure 4, region III) was not significantly different by age (p > 0.1).

The inclusion of a seroreversion rate in the model also slightly increased the estimated seroconversion rates. Due to the cross-sectional nature of this study and the long duration of antibody detection for AMA-1 and MSP-1, it was appropriate to set the seroreversion rate to zero. When seroreversion was included in the model, the seroreversion rates were -0.006 (95% CI -0.016, 0.003) and 0.008 (95% CI -0.005, 0.022) for AMA-1 and MSP-1 respectively. Since both of the confidence intervals for the seroreversion rates include zero and the inclusion of a seroreversion rate in the model had little effect on the estimates of seroconversion, therefore *a priori* exclusion of a seroreversion rate from the final model was justified in this circumstance. When comparing the estimated seroconversion rates from study participants under 20 years of age, the continuity in the age-specific seroprevalence curve could indicate that over multiple decades, a relatively constant state of low malaria transmission has occurred in these regions, even in the absence of sustained malaria control efforts. Entomological studies investigating the vector competency of A. albimanus mosquito, may better explain this phenomenon, of stable low transmission.

### Limitations

One of the primary limitations of this study was that serum samples were collected using a convenience sample, which limited our ability to infer how this sample population represents Haiti as a whole. Findings may have been skewed by potentially enrolling participants from clinics (n = 203), however, this potential sampling bias was adequately addressed by excluding all malaria RDT positive individuals (5/815) from final analysis. This study also only screened for previous exposure to *P. falciparum*, although the likelihood of finding other species or mixed infections remains low, given recent reports, and the presence of host protective factors [5, 20]. As with other ELISA protocols, setting a threshold for the classification of a sample as seropositive is subject to interpretation. To validate the method used in this study, thresholds using absorbance values of four and five standard deviations above the suspected seronegative population mean were also evaluated and fell within the calculated confidence intervals for seroprevalence and SCR using only three standard deviations.

## Conclusion

As reported cases of malaria in the Dominican Republic have reached a 15-year low of 952 cases [1], malaria continues to be a major public health concern in Haiti. Findings from this study further support the notion of sustained low low-level transmission in Haiti, while using a highly sensitive technique that could be used to determine malaria transmission elsewhere in Haiti. These data suggests that any efforts to advance malaria control locally have not had much impact over the last five decades, yet neither have the past political upheavals or natural disasters from recent decades resulted in major malaria epidemics.

Future studies should expand seroprevalence methodologies to other departments in order to establish countrywide trends. Research examining barriers to access, protective host characteristics, the extent of heterogeneous malaria transmission in other departments, and vector proficiency could also enhance elimination models in Haiti. Elimination in Haiti appears to be feasible; however, surveillance must continue to be strengthened in order to respond to areas with high transmission, while measuring the impact of future interventions.
